# Bullous Pemphigoid Disease Area Index Pruritus, Activity, and Damage During Biologic Therapy in Bullous Pemphigoid: An International Dual‐Center Study

**DOI:** 10.1111/1346-8138.70389

**Published:** 2026-07-12

**Authors:** Ruggero Cascio Ingurgio, Angela Alfano, Aalia Syed, Luigi Gargiulo, Alessandra Narcisi, Antonio Costanzo, Dedee F. Murrell

**Affiliations:** ^1^ Dermatology Unit IRCCS Humanitas Research Hospital Rozzano Milan Italy; ^2^ Department of Biomedical Sciences Humanitas University Pieve Emanuele Milan Italy; ^3^ Faculty of Medicine University of New South Wales Sydney New South Wales Australia; ^4^ Department of Dermatology St George Hospital Sydney New South Wales Australia

**Keywords:** biological therapy, bullous pemphigoid, follow‐up studies, pruritus, treatment outcome

## Abstract

Biologic therapies targeting Type 2 inflammation are increasingly used in bullous pemphigoid, yet the relationship between pruritus and distinct disease domains remains incompletely defined. We conducted a retrospective international dual‐center study of 25 patients with bullous pemphigoid treated with dupilumab, omalizumab, or tralokinumab. Bullous Pemphigoid Disease Area Index activity, damage, and pruritus were assessed at baseline and at Weeks 8, 16, 32, and 52. Mixed‐effects models showed significant improvement in disease activity and pruritus from Week 8 onward, whereas damage showed a slower trajectory, becoming significant only at later timepoints. Pruritus improvement correlated with improvement in disease activity across all timepoints. Clinical response rates increased over follow‐up. Although systemic corticosteroids were frequently used at treatment initiation and tapered thereafter, improvement was sustained during corticosteroid weaning. These findings support pruritus as a dynamic and clinically meaningful domain for monitoring treatment response, linked to inflammatory disease activity rather than residual damage.

## 1 Introduction

Bullous pemphigoid (BP) is the most common autoimmune blistering disease, characterized by intense pruritus and inflammatory skin involvement in elderly patients [1]. Systemic corticosteroids remain the mainstay of treatment but are associated with substantial toxicity, particularly in frail patients [2]. Consequently, biologic therapies targeting type 2 inflammatory pathways, including dupilumab and omalizumab, are increasingly used as steroid‐sparing strategies in clinical practice [3, 4, 5].

Real‐world studies support biologic efficacy in BP but have mainly focused on global disease control rather than specific disease domains [4, 5, 6, 7]. The Bullous Pemphigoid Disease Area Index (BPDAI) distinguishes activity, damage, and pruritus, allowing detailed evaluation of treatment response [8, 9]. Whether pruritus improvement reflects reduction in inflammatory activity or residual damage remains unclear. We evaluated longitudinal trajectories of BPDAI‐activity, damage, and pruritus in BP patients treated with biologics.

## 2 Methods

We performed a retrospective observational study of consecutive patients with BP treated with biologic therapy at Humanitas Research Hospital (Milan, Italy) and St George Hospital (Sydney, Australia). The diagnosis of bullous pemphigoid was based on clinicopathological and immunological findings. Patients received dupilumab, omalizumab, or tralokinumab as off‐label therapies for bullous pemphigoid in routine clinical practice. Biologics were administered according to approved dosing regimens for atopic dermatitis or chronic spontaneous urticaria and prescribed off‐label according to physician judgment, mainly for steroid‐sparing purposes, inadequate disease control, or intolerance/contraindications to conventional therapies. No standardized treatment or tapering protocol was applied. Biologic selection, corticosteroid tapering, and concomitant therapies were individualized according to clinical response and patient characteristics. BPDAI‐activity, BPDAI‐damage, and BPDAI‐pruritus were recorded at baseline and at Weeks 8, 16, 32, and 52 when available. Baseline demographic and clinical characteristics are summarized in Table 1.

**TABLE 1:** Baseline demographic and clinical characteristics of the study population.

Characteristic	Total (*n* = 25)
Age, years	79 (68–87)
Male, *n* (%)	13 (52%)
BMI, kg/m^2^	30.7 (23.9–42.4)
Age at BP onset, years	74 (61–82)
Mucosal involvement, *n* (%)	1 (4%)
BPDAI activity at baseline	20 (7–28)
BPDAI damage at baseline	2 (0–7)
BPDAI‐pruritus at baseline	20 (10–23)
Number of comorbidities	3 (1–4)
**Treatment characteristics**	
Dupilumab, *n* (%)	20 (80%)
Omalizumab, *n* (%)	4 (16%)
Tralokinumab, *n* (%)	1 (4%)
Previous topical corticosteroids, *n* (%)	23 (92%)
Previous systemic corticosteroids, *n* (%)	22 (88%)
Prednisone dose at baseline, mg/day	10 (0–25)
Previous immunosuppressants, *n* (%)	10 (40%)
Previous tetracycline, *n* (%)	8 (32%)
Previous biologics, *n* (%)	3 (12%)
**Comorbidities**	
Arterial hypertension, *n* (%)	18 (72%)
Type 2 diabetes, *n* (%)	7 (28%)
Dyslipidemia, *n* (%)	12 (48%)
Cardiovascular disease, *n* (%)	8 (32%)
COPD/asthma, *n* (%)	2 (8%)
Malignancy, *n* (%)	3 (12%)
Non‐melanoma skin cancer, *n* (%)	1 (4%)
Bone disease, *n* (%)	4 (16%)
Thyroid disease, *n* (%)	2 (8%)

*Note:* Values are expressed as median (interquartile range) unless otherwise indicated.

Abbreviations: BP, bullous pemphigoid; BPDAI, Bullous Pemphigoid Disease Area Index; BMI, body mass index; COPD, chronic obstructive pulmonary disease.

Twenty‐five patients were included: most patients received dupilumab (*n* = 20), while four received omalizumab and one tralokinumab. Previous immunosuppressants included methotrexate (*n* = 7), mycophenolate mofetil (*n* = 2), and azathioprine (*n* = 1). Three patients had previously received biologics (rituximab, *n* = 2; efgartigimod, *n* = 1). Follow‐up data were available for 25 patients at Week 8, 25 patients at Week 16, 22 patients at Week 32, and 16 patients at Week 52. Patients frequently received systemic corticosteroids at treatment initiation, progressively tapered as clinical improvement occurred. Continuous variables are reported as median and interquartile range (IQR), and categorical variables as number/total (%). Statistical analyses were performed using StataNow/SE 19.5 (StataCorp, College Station, TX, USA). Longitudinal BPDAI changes were analyzed using mixed‐effects linear models with time as a fixed effect and patient as random intercept. Comparisons versus baseline were performed using Sidak adjustment for multiple testing. Clinical response was defined as a ≥ 50% reduction from baseline in BPDAI‐activity or BPDAI‐pruritus. Patients with a baseline score of zero were excluded from percentage‐response analyses because relative change could not be calculated. Spearman correlation was used for association analyses. Missing data were not imputed. Analyses used all available observations. A two‐sided *p* value < 0.05 was considered statistically significant. The study was conducted in accordance with institutional ethical standards and the Declaration of Helsinki.

## 3 Results

Median BPDAI‐activity decreased from 20 (7–28) at baseline to 7 (0–9) at Week 8, 4 (0–9) at #16, 0.5 (0–4) at Week 32, and 0 (0–4) at Week 52. In mixed‐effects models accounting for repeated measurements and unbalanced follow‐up, BPDAI‐activity was significantly reduced compared with baseline at Week 8, Week 16, Week 32, and Week 52 (all Sidak‐adjusted *p* < 0.001).

BPDAI‐pruritus decreased from 20 (10–23) at baseline to 10 (0–12) at Week 8, 6 (2–14) at Week 16, 4.5 (0–10) at Week 32, and 0 (0–7.5) at Week 52. Mixed‐effects analysis showed significant reductions at Week 8 (Sidak‐adjusted *p* = 0.002) and persisting through Weeks 16, 32, and 52 (all Sidak‐adjusted *p* < 0.001).

**FIGURE 1:**
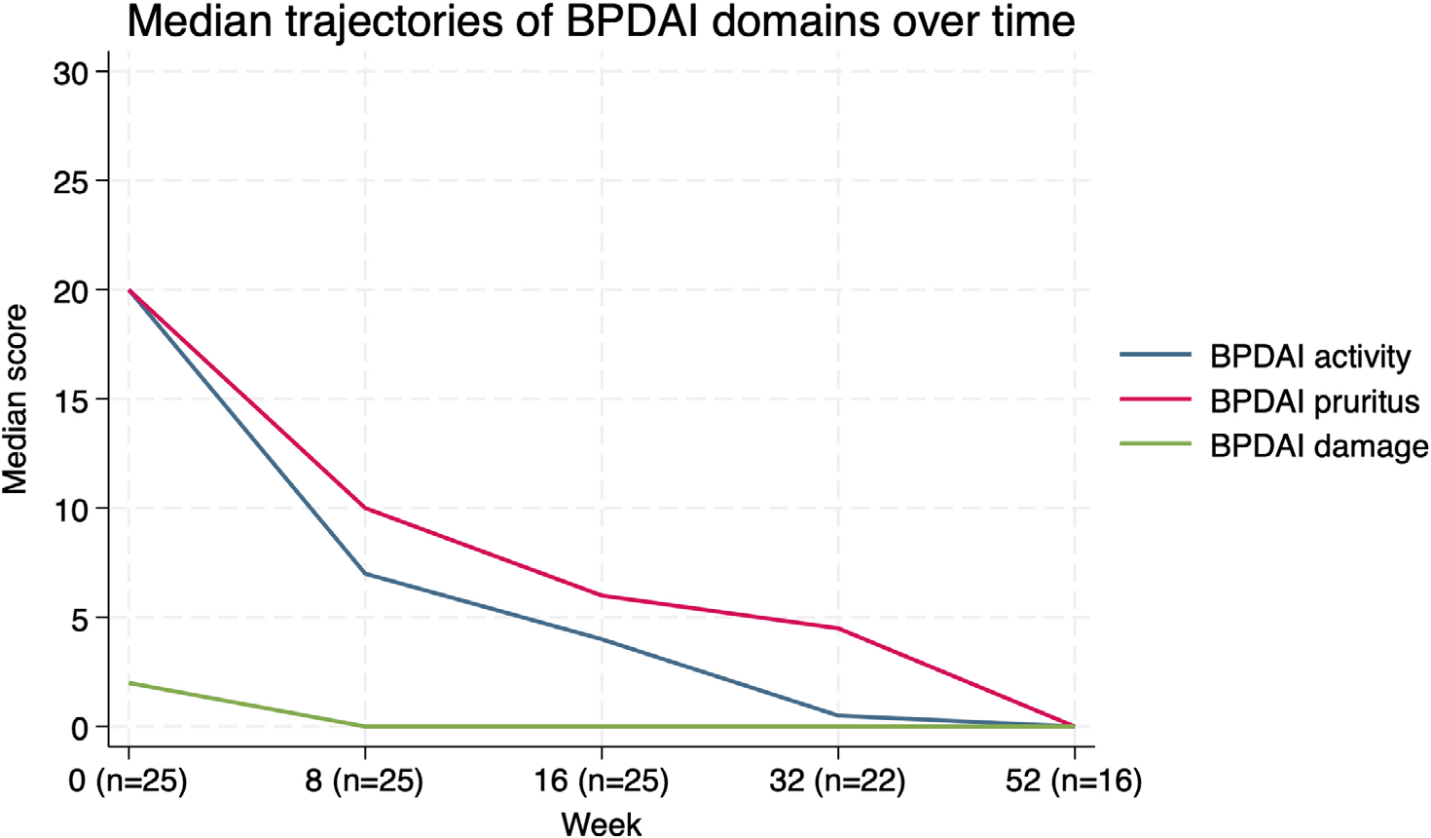
Longitudinal evolution of BPDAI domains during biologic therapy. Median values of BPDAI‐activity, BPDAI‐damage, and BPDAI‐pruritus at baseline and during follow‐up at Weeks 8, 16, 32, and 52.

BPDAI‐damage showed a slower trajectory, decreasing from 2 (0–7) at baseline to 0 (0–5) at Week 8, 0 (0–4) at Week 16, 0 (0–4) at Week 32, and 0 (0–2) at Week 52. In mixed‐effects models, reductions in BPDAI‐damage were not significant at Weeks 8 and 16 after Sidak adjustment but became significant at Week 32 (*p* = 0.007) and Week 52 (*p* = 0.012). BPDAI trajectories are shown in Figure 1.

Clinical response rates increased. A ≥ 50% reduction from baseline in BPDAI‐activity was observed in 15/24 patients (62.5%) at Week 8, 18/25 patients (72.0%) at Week 16, 17/22 (77.3%) at Week 32, and 13/16 (81.3%) at Week 52. BPDAI‐pruritus response rates were 7/25 (28.0%), 14/25 (56.0%), 16/22 (72.7%), and 12/16 (75.0%), respectively, suggesting a slightly delayed symptomatic response.

Systemic corticosteroid exposure decreased significantly over time. Median prednisone‐equivalent dose declined from 10 mg/day (0–25) at baseline to 5 (0–12.5) mg/day at Week 8, 0 (0–6) mg/day at Week 16, 0 (0–5) mg/day at Week 32, and 0 (0–0) mg/day at Week 52. The proportion of patients who had discontinued systemic corticosteroids increased from 8/25 (32.0%) at Week 8 to 13/25 (52.0%) at Week 16, 16/22 (72.7%) at Week 32, and 13/16 (81.3%) at Week 52.

Mild adverse events included dyspnea, fatigue, flu‐like illness, and myalgia, without treatment discontinuation.

**FIGURE 2:**
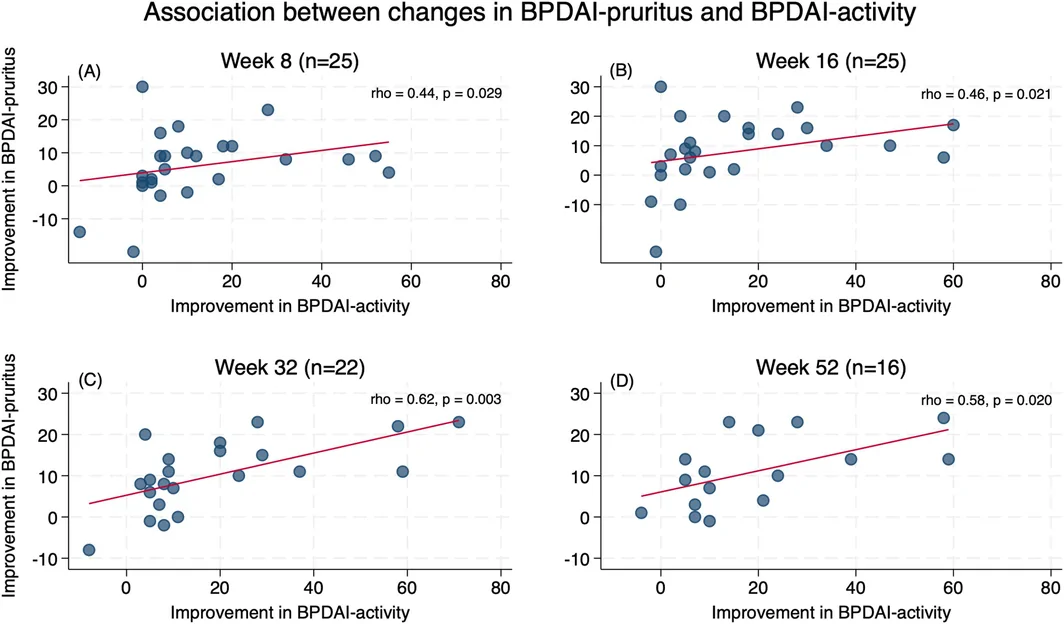
Association between changes in BPDAI‐pruritus and BPDAI‐activity over time. (A) Week 8, (B) Week 16, (C) Week 32, and (D) Week 52. Scatter plots show the relationship between improvement in BPDAI‐activity and BPDAI‐pruritus at each time point, with fitted linear regression lines. Improvement was calculated as baseline score minus follow‐up score, with positive values indicating improvement and negative values indicating worsening. Spearman correlation coefficients and *p* values are reported within each panel.

Improvement in BPDAI‐pruritus correlated significantly with improvement in BPDAI‐activity at Week 8 (rho = 0.44, *p* = 0.029), Week 16 (rho = 0.46, *p* = 0.021), Week 32 (rho = 0.62, *p* = 0.003), and Week 52 (rho = 0.58, *p* = 0.020). By contrast, no significant association was observed between BPDAI‐pruritus improvement and BPDAI‐damage at Week 8 (rho = 0.20, *p* = 0.329), Week 16 (rho = 0.18, *p* = 0.391), Week 32 (rho = 0.03, *p* = 0.891), or Week 52 (rho = −0.20, *p* = 0.462). These findings indicate that BP‐specific pruritus improvement parallels reduction in disease activity rather than damage. Scatter plots are shown in Figure 2A–D.

Reduction in corticosteroid dose correlated with improvement in BPDAI‐activity at Week 8 (Spearman's rho = 0.65, *p* = 0.0007), at Week 16 (rho 0.61, *p* = 0.0016) and Week 32 (rho 0.52, *p* = 0.0146), and with improvement in BPDAI‐pruritus at Week 32 (rho 0.61, *p* = 0.0030), while correlations at Week 52 were not significant.

## 4 Discussion

In this dual‐center cohort, biologic therapy was associated with early and sustained improvement in BP disease activity and pruritus over follow‐up. The most relevant finding is that BPDAI‐pruritus behaved as a dynamic domain linked to BPDAI‐activity, whereas BPDAI‐damage appeared less informative of symptomatic response [10]. Damage reflects residual or post‐inflammatory changes that may persist despite inflammatory control.

Activity and pruritus improved early, with significant reductions already detectable at Week 8 in mixed‐effects analyses, and response rates increased progressively over follow‐up. Pruritus responses were lower than activity responses at Week 8 and Week 16, but converged later, suggesting a slightly delayed symptomatic response. This variability suggests that pruritus may improve more slowly than disease activity in some patients early in treatment. Correlation analyses (Figure 2A–D) showed that pruritus improvement was associated with activity improvement but not with damage.

Corticosteroid doses were progressively reduced over time, with many patients achieving discontinuation during follow‐up, supporting a steroid‐sparing effect of biologic therapy. Reduction in corticosteroid dose paralleled improvement in BPDAI‐activity and pruritus. However, given the observational design and the concomitant use of corticosteroids at treatment initiation, we cannot exclude that part of the early clinical response was influenced by systemic corticosteroids, and causality cannot be definitively established.

Limitations include the retrospective design, small sample size, attrition over time, and treatment‐group imbalance, precluding meaningful comparative analyses across biologics. The low damage scores observed at later timepoints may also reflect a floor effect, limiting the ability of damage to discriminate residual disease burden. The absence of a control group precludes causal inference regarding biologic efficacy and does not allow separation of the effects of biologic therapy from concomitant corticosteroid tapering or the natural disease course. Although pairwise comparisons in mixed‐effects models were adjusted using the Sidak method, other secondary analyses were exploratory and should be interpreted cautiously. Separate BPDAI activity components and biomarkers potentially related to pruritus, such as eosinophil count and total IgE, were not systematically available and could not be analyzed.

Despite these limitations, serial BPDAI measurements provide insight into BP evolution during biologic therapy. BP‐specific pruritus may represent a dynamic outcome reflecting inflammatory activity rather than damage. This supports its clinical relevance for monitoring treatment response.

In conclusion, biologic therapy in BP was associated with early and sustained improvement in BPDAI‐activity and BPDAI‐pruritus, whereas BPDAI‐damage followed a slower trajectory. Pruritus improvement correlated with activity but not with damage, supporting domain‐based assessment of treatment response.

## Ethics Statement

The authors have nothing to report.

## Consent

The requirement for written informed consent was waived due to the retrospective nature of the study, in accordance with local regulations.

## Conflicts of Interest

R. Cascio Ingurgio has served as a consultant for UCB Pharma. L. Gargiulo has been a consultant and/or speaker and has participated in advisory boards for AbbVie, Almirall, Eli Lilly, Pfizer, Sanofi and UCB Pharma. A. Narcisi has served on advisory boards, received honoraria for lectures and research grants from Almirall, AbbVie, Leo Pharma, Celgene, Eli Lilly, Janssen, Novartis, Sanofi‐Genzyme, Amgen and Boehringer Ingelheim. A. Costanzo has served as an advisory board member, consultant and has received fees and speaker's honoraria or has participated in clinical trials for AbbVie, Almirall, Biogen, LEO Pharma, Lilly, Janssen, Novartis, Pfizer, Sanofi Genzyme and UCB Pharma. D.F. Murrell is an author for UpToDate for BP, has scientific cooperations and advisory roles in the field of eczema and psoriasis with Sanofi, AbbVie, Arena, Attovia, BMS, UCB, Incyte, Leo, Lilly, Galderma, Dermira, Pfizer, BMS, Kineska, Fujisawa, Novartis and Evelo. In the area of blistering diseases, she has scientific cooperations and advisory roles with ArgenX, Sanofi Regeneron, Principia, Janssen, Lilly, Ono, Almirall, RAPT and Roche. She is creator of the ABQOL/TABQOL and co‐creator of the BPDAI. The other authors declare no conflicts of interest.

## Data Availability

The data that support the findings of this study are available from the corresponding author upon reasonable request.
